# The crystal structure of olanzapine form III

**DOI:** 10.1107/S2052252524007383

**Published:** 2024-07-29

**Authors:** Goulielmina Anyfanti, Elena Husanu, Iryna Andrusenko, Danilo Marchetti, Mauro Gemmi

**Affiliations:** a Electron Crystallography Istituto Italiano di Tecnologia (ITT) Viale Rianaldo Piaggio 34 Pontedera56025 Italy; b Department of Chemistry University of Parma Parco Area delle Scienze 17/A Parma43124 Italy; University of California, Los Angeles, USA

**Keywords:** 3D electron diffraction, 3D ED, olanzapine, organic crystallography, *ab initio* structure determination, dynamical refinement

## Abstract

The antipsychotic drug olanzapine is well known for its complex polymorphism. Although widely investigated, the crystal structure of one of its anhydrous polymorphs, form III, is still unknown. Its appearance, always in concomitance with forms II and I, and the impossibility of isolating it from that mixture, have prevented its structure determination so far. The scenario has changed with the emerging field of 3D electron diffraction (3D ED) and its great advantages in the characterization of polyphasic mixtures of nanosized crystals. In this study, we show how the application of 3D ED allows the *ab initio* structure determination and dynamical refinement of this elusive crystal structure that remained unknown for more than 20 years. Olanzapine form III is monoclinic and shows a similar but shifted packing with respect to form II. It is remarkably different from the lowest-energy structures predicted by the energy-minimization algorithms of crystal structure prediction.

## Abstract 2

This paper reports the crystal structure determination of olanzapine form III by 3D electron diffraction, the last unknown anhydrous polymorph in the olanzapine complex polytypism scenario.

## 1. Introduction

**Figure 1:**
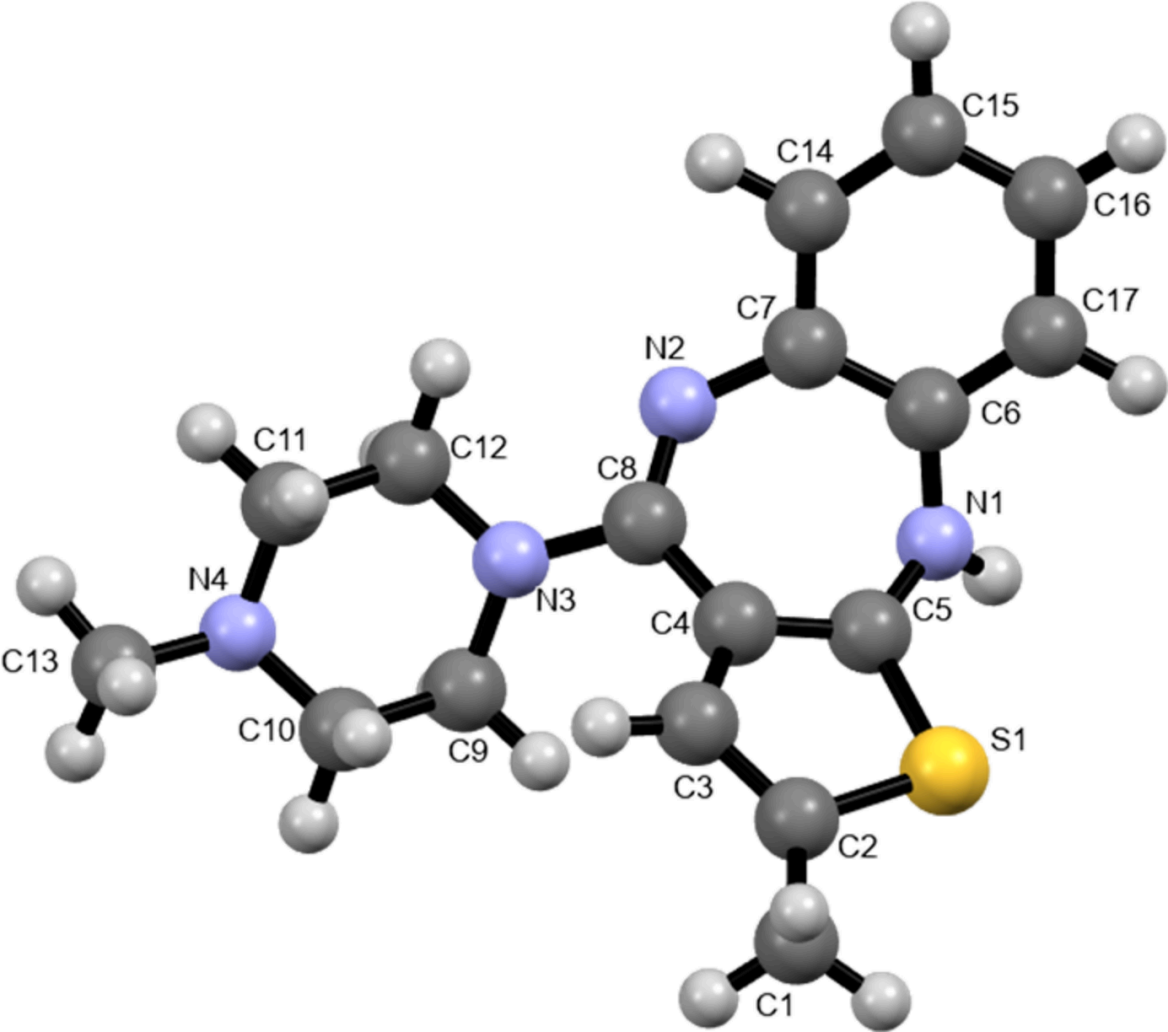
Molecular structure of olanzapine.

Olanzapine (OLZP) (Fig. 1 ▸), a thienobenzodiazepine derivative, is a second-generation antipsychotic mostly used for the treatment of schizophrenia and bipolar disorder owing to its antagonist action for multiple neurotransmitter receptor sites (Saadatjoo *et al.*, 2016 ▸).

Its ability to assume different solid-state phases forming anhydrous, hydrated and solvated polymorphs (Reutzel-Edens *et al.*, 2003 ▸; Wawrzycka-Gorczyca *et al.*, 2004 ▸; Thakuria & Nangia, 2011 ▸; Bhardwaj *et al.*, 2013 ▸; Askin *et al.*, 2019 ▸; Reutzel-Edens & Bhardwaj, 2020 ▸; Valdivia-Berroeta *et al.*, 2021 ▸; Ayala *et al.*, 2006 ▸; Tang *et al.*, 2021 ▸) highlights the reason for such intense research work around its polymorphism. In the pharmaceutical industry, the control of possible solid forms and, therefore, of the physicochemical properties such as bioavailability, solubility and stability is the basic requirement in drug development (Burger & Ramberger, 1979 ▸; Brittain, 1999 ▸; Rodríguez-Spong, 2004 ▸; Cruz-Cabeza & Bernstein, 2014 ▸; Lee, 2014 ▸) and a key point of patenting. (Bernstein, 2002 ▸)

OLZP has more than 60 polymorphs, only four of which are known to be anhydrous phases. Three of these, forms I, II and III, were discovered during the development of the marketed pharmaceutical Zyprexa at Lilly research laboratories (Reutzel-Edens & Bhardwaj, 2020 ▸) and resulted in a patent which dates back to 1993 (Chakrabarti *et al.*, 1993 ▸). Form I has been confirmed as thermodynamically stable and was selected for the commercial development into Zyprexa (Reutzel-Edens & Bhardwaj, 2020 ▸). It is the only form crystallized as a single phase by direct crystallization from solvents that do not form solvates with OLZP. Forms II and III can be crystallized as desolvation products of certain OLZP solvates (Ayala *et al.*, 2006 ▸) but, until now, could not be isolated as pure polycrystalline phases. Both forms I and II can be grown as single crystals large enough for single-crystal X-ray diffraction, so their structures have been determined (Reutzel-Edens *et al.*, 2003 ▸; Thakuria & Nangia, 2011 ▸; Bhardwaj *et al.*, 2013 ▸). To the best of our knowledge, all the efforts of different research groups to obtain OLZP form III in pure form have failed, and the concomitant appearance of phase III with phase II and its microcrystalline character have prevented its structure determination so far. A possible structure has been proposed via crystal structure prediction (CSP) techniques (Bhardwaj *et al.*, 2013 ▸; Leblanc & Johnson, 2019 ▸), but a trial to fit powder X-ray diffraction with a two phases Pawley-type refinement was not fully satisfactory since some peaks belonging to phase III were not properly modelled. Both phases I and II, as well as the proposed structural model of phase III, which is an alternative layer stacking of phase II, are based on a ‘dispersion’ dimer of opposite enantiomer pairs related by a centre of symmetry identified as the supramolecular construct SC_0_ by Bhardwaj *et al.* (2013 ▸). The dimer is held together by dispersion forces and the two enantiomers have exposed hydrogen-bond donors and acceptors on the outer part which are responsible for the crystal structure packing. Interestingly, the CSP study that delivered the only available structural model of phase III proposed also, among others, some OLPZ structures in which the SC_0_ was absent. This triggered a dedicated search of new OLPZ polymorphs through non-conventional crystallization methods such as polymer based molecular dispersion, which resulted in the discovery of the new anhydrous form IV (Askin *et al.*, 2019 ▸). In this scenario, the crystal structure of phase III is, for the moment, the only missing tile in the puzzle of anhydrous OLZP polymorphism. Although crystalline structural changes are generally monitored through single-crystal and powder X-ray diffraction analysis to gain an overview of the molecular arrangement and their relationship with bulk properties, the inherent limitations of OLZP phase III challenge the two complementary techniques in its structure determination. In recent years, many studies have shown the effectiveness of single-crystal electron diffraction, known as 3D ED or MicroED (Gemmi *et al.*, 2019 ▸), in elucidating the structure solution of organic nanocrystals of pharmaceutical interest (Jones *et al.*, 2018 ▸; Andrusenko *et al.*, 2019 ▸, 2021 ▸; Gruene & Mugnaioli, 2021 ▸; Andrusenko & Gemmi, 2022 ▸; Li *et al.*, 2023 ▸). For these reasons, and the importance of the structure dilemma of OLPZ phase III, we decided to examine the powder mixture of OLZP forms II and III with 3D ED and, surprisingly, were able to solve the crystal structure of the latter *ab initio*. Dynamical refinements led us, on one hand, to the final model of form III that showed a different packing of layers with respect to form II. On the other hand, the structure of OLPZ form III was solved in *P*2_1_/*c* and not *Pbca*, differing from the predicted model (Bhardwaj *et al.*, 2013 ▸).

## 2. Results and discussion

Starting from a pure OLZP form I sample [see the Le Bail fit in Fig. S1 of the supporting information (Le Bail, 2005 ▸)], a mixture of OLZP forms I, II and III with a higher content of the latter was obtained using a slightly modified method of crystallization than that published by Reutzel-Edens *et al.* (2003 ▸) (see the supporting information). The characteristic powder pattern and differential scanning calorimetry (DSC) thermograms (Figs. S2 and S3) of the mixture coincide with that observed by Bhardwaj *et al.* (2013 ▸). The sample, under a transmission electron microscope (TEM), revealed the presence of micro- to nanosized crystals. Although crystal habit prediction studies (Luo *et al.*, 2019 ▸; Lu *et al.*, 2021 ▸) have described how OLZP crystals of the same polymorph can adopt different morphologies depending on the crystallization solvent used (Lu *et al.*, 2021 ▸), all the grains observed under a TEM show an irregular shape and any distinction based on the habit of the nanosized crystal was impossible (Fig. S4). 3D ED data were collected over 32 crystals and their indexing confirmed the presence of three different polymorphs. A first group of crystals which could be indexed as form II, a second very rare group which could be indexed as form I and a third group which could be indexed with a monoclinic cell not yet reported. We considered the latter as a representative of form III (CCDC No. 2309728 contains the supplementary crystallographic data for this paper, available free of charge by the joint Cambridge Crystallographic Data Centre and Fachinformationszentrum Karlsruhe Access Structures service). Since form I crystals were rare and diffracted only at low resolution, we identified them only through their unit-cell determination. The structure of both forms II and III could be solved *ab initio* instead. Table 1 ▸ shows the unit-cell parameters of the three forms along with the crystallographic data and refinements for phases III and II.

The corresponding images of the *hk*0, *h*0*l* and 0*kl* reciprocal space sections are shown in Fig. S5. The structure of form II was solved and kinematically refined and corresponds to the one reported in the literature, whereas form III is new and differs from the predicted model A162. Since form II is known and single-crystal X-ray refinements are available, we decided not to proceed further in its structure analysis. However, even for OLZP form II we performed a dynamical refinement against one dataset. The results are listed in Table S1 in the supporting information. Form III crystallizes in the monoclinic space group *P*2_1_/*c* as form II but with a larger monoclinic angle of 110° instead of 98°. The quality of the collected 3D ED data prompted us to refine the form III structural model considering the dynamical diffraction theory (Palatinus *et al.*, 2019 ▸; Petříček *et al.*, 2023 ▸). Dynamical refinement takes into account the multiple scattering effects delivering more precise structures and fitting much better the experimental intensities. Still, not a diffuse practice in the case of unknown organic structures, the use of dynamical refinement results in a drop in the residual *R* value which usually reduces to half with respect to kinematical refinements. The latter are reported in Table S2. In our case, the solved structure of form III was dynamically refined against two datasets at a resolution of 1 Å, reaching a final *R* value of 12.23%. The thickness variation during crystal rotation was modelled with the thick model wedge approximation (Palatinus *et al.*, 2015 ▸) and the final calculated thicknesses for the two crystals are 129 and 120 nm, respectively. Atomic displacement parameters (ADPs) of all non-hydrogen atoms were refined anisotropically. Atoms in a similar chemical environment were restrained to have similar anisotropic ADPs. For instance, the anisotropic ADPs of C15, C16 and C17 atoms were kept similar to that of C14. The 3D ED data quality is sensitive to the presence of hydrogens (Palatinus *et al.*, 2017 ▸). Though some of them can be already identified in the difference Fourier map before the refinement (Fig. S6), neglecting the hydrogens significantly worsens the refinement. This can be checked, not only by the *R* value that drops from 15.77% (*R*_obs_) to 12.23% (*R*_obs_), but also by the difference Fourier map that, once aligned and scaled to the same isosurface value of 0.27 e Å^−1^ using *VESTA* (Momma & Izumi, 2011 ▸), is much less noisy after the hydrogen addition than before (Fig. S7).

**Figure 2:**
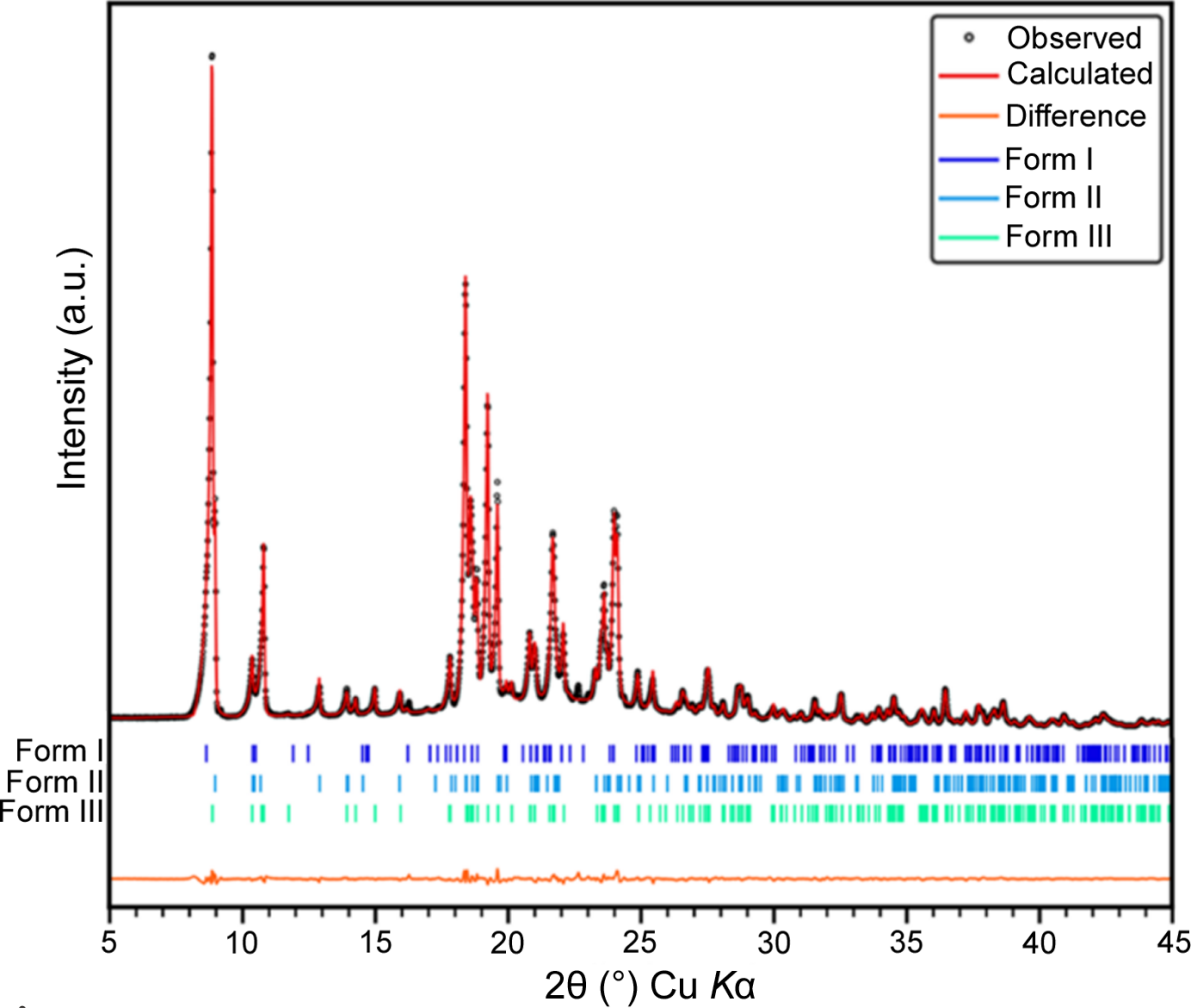
Three-phase Rietveld plot of the multiphase powder sample.

A Le Bail fit of the powder pattern (Fig. S8) collected on the sample confirmed the mixture of the three forms, while a Rietveld refinement (Fig. 2 ▸, *R*_obs_ = 3.11%, *wR*_obs_ = 4.26%) further assures that the structural model of form III derived from 3D ED is correct.

The Rietveld refinement also states that form III is the major phase with a volume fraction of 68% followed by form II with a volume fraction of 25.8%, and an almost negligible amount of form I (6.2%), in agreement with previous observations (Bhardwaj *et al.*, 2013 ▸).

**Figure 3:**
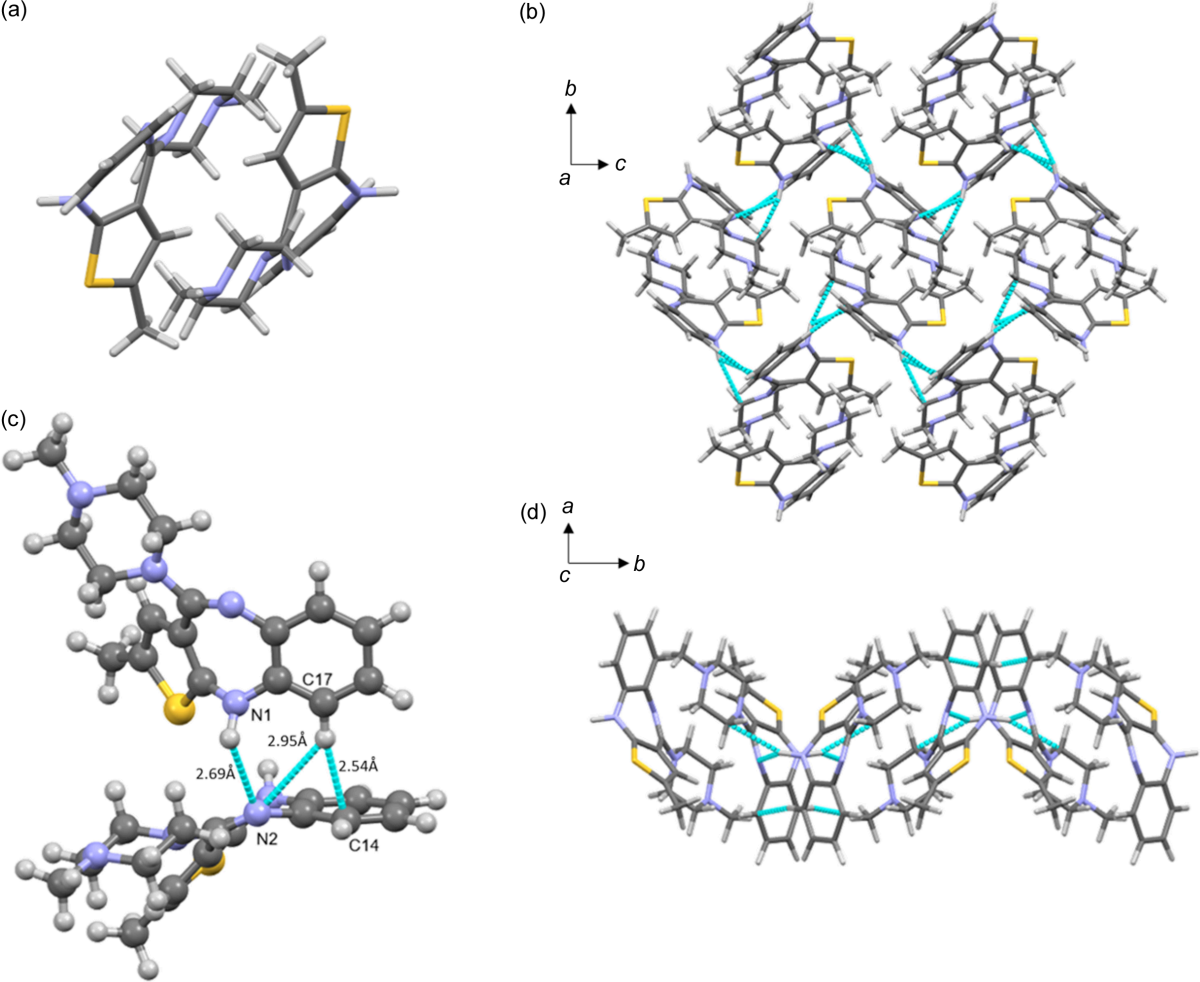
(*a*) Head-to-tail enantiomers forming the centrosymmetric building block SC_0_ of OLZP form III. (*b*) 2D layer of OLZP form III viewed along the *a* axis with the relative intermolecular interactions among adjacent SC_0_ dimers. (*c*) Closer view of the intermolecular contacts between two adjacent molecules with relative distances of 2.69 Å for the N1H⋯N2 contact, 2.95 Å for the C17H⋯N2 contact and 2.54 Å for the C17H⋯C14 contact. (*d*) 2D corrugated plane of OLZP along the *c* axis with the relative intermolecular contact network.

The characteristic building block of phase III is the same face-to-face centrosymmetric dimer motif SC_0_ (Bhardwaj *et al.*, 2013 ▸) [Fig. 3 ▸(*a*)] encountered in forms I and II and also in the predicted structure of form III, the orthorhombic A162. It consists of head-to-tail enantiomers bound by only dispersion interactions. Each enantiomer assumes the same conformation observed and described by Reutzel-Edens *et al.* (2003 ▸), where the piperazine ring in its chair conformation is almost coplanar with the puckered diazepine ring. Each SC_0_ dimer interacts with the adjacent ones through dispersive interactions and a unique hydrogen-bonded network [Fig. 3 ▸(*b*)]. Inside this network, we can distinguish an NH⋯N interaction between N1, as a hydrogen-bond donor, and the neighbouring N2 (*x*, 3/2 − *y*, −1/2 + *z*), as a hydrogen-bond acceptor. Another weaker intermolecular interaction that contributes to the packing stability of OLZP form III is a bifurcated contact between C17—H, as a hydrogen-bond donor, and N2 (*x*, 3/2 − *y*, −1/2 + *z*) or C14 (*x*, 3/2 − *y*, −1/2 + *z*) as a hydrogen-bond acceptor (CH⋯π) [Fig. 3 ▸(*c*)]. As in form II, in phase III the bonding network drives the formation of corrugated planes parallel to (100) that, if viewed along the *c* direction, exhibit a wavy shape [Fig. 3 ▸(*d*)].

Up to now, we have dealt with the arrangement of the intermolecular interactions of form III by considering the number of atom–atom contacts that have the greatest effect on the stability of the crystal, for instance the hydrogen bonds. The whole-to-molecule approach adopted in the calculation of the Hirshfeld isosurfaces (Spackman & Byrom, 1997 ▸; McKinnon *et al.*, 1998 ▸; Spackman & Jayatilaka, 2009 ▸) gives, instead, a global visualization of the type of short contacts involved in the crystal packing.

**Figure 4:**
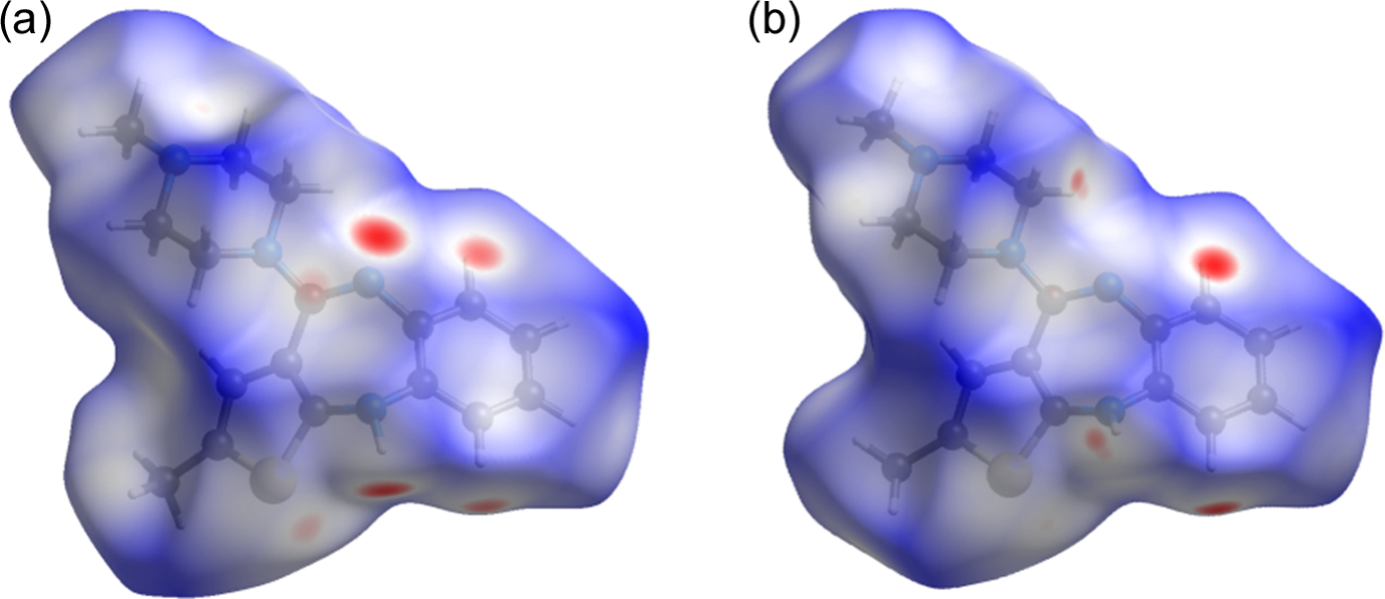
Hirshfeld surface of an OLZP molecule in forms (*a*) II and (*b*) III.

Fig. 4 ▸ compares the Hirshfeld surfaces calculated for forms II and III. The red areas highlight the close contacts of the molecule with the surroundings and, in both phases, they coincide with what has already been described. The sulfur atom of the thio­phene ring also forms a close contact with neighbouring molecules. The relative contribution of each intermolecular interaction on each surface is shown in the fingerprint graphs (Spackman & McKinnon, 2002 ▸; McKinnon *et al.*, 2007 ▸) of the two surfaces in Fig. S9 as well as in Table S3.

**Figure 5:**
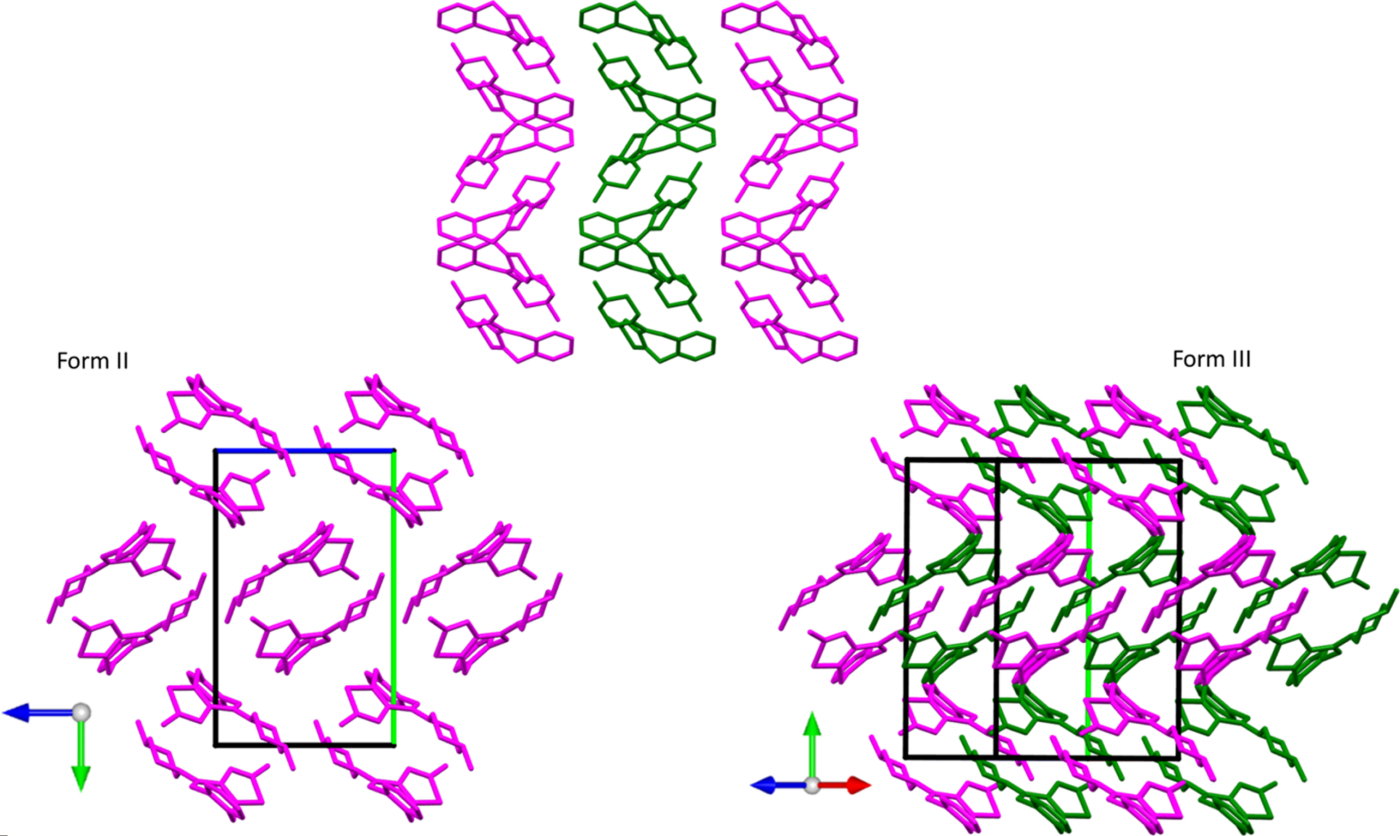
(Top) Packing of the three layers in forms II and III, viewed along the relative *c* axis, the central layer is green for the visualization of the different packing; the two structures are rotated and viewed along the *a* axis in form II (bottom left) and along the equivalent crystallographic axis in the monoclinic structure found of phase III where the shift of the pink layers is evident (bottom right). The unit-cell axes are coloured red (*a*), green (*b*) and blue (*c*).

In all forms, the dispersion dimer is the unit that, repeated along a plane (*bc* in form II and III, and *ac* in A162), forms 2D layers. The main difference between forms II, III and the predicted form A162 arises in the way that the 2D layers are packed in the third dimension. To better understand and display this difference, we first compared the structure of form II with our model of form III. Fig. 5 ▸ (top) shows the stacking of three consecutive layers viewed along the *c* axis for forms II and III, coloured alternatively in green and pink. Looking at the structure perpendicular to these layers and taking as a reference the orientation of the dimers viewed along the *a* axis of form II, the difference in the stacking can be clearly highlighted. In form II, all the dimers of the different layers perfectly superimpose (only the pink ones are visible) (Fig. 5 ▸ bottom left), whereas in form III we can observe a clear shift of the green and pink levels along *c* (Fig. 5 ▸ bottom right), which is responsible for the increase in the β angle of nearly 12° between the two structures.

The displacement along the third dimension is the main difference claimed also by Bhardwaj *et al.* (2013 ▸) in A162, but, if in form III there is a shift along one direction every second layer, in A162 the shift is obtained through a *c* glide perpendicular to *b*, which causes the exchange of the enantiomers every second layer. For a better understanding, see Fig. S10. A careful screening of the CSP structures of OLZP obtained by Bhardwaj *et al.* (2013 ▸) and kindly provided by the authors lead us to take into consideration one predicted structure – UNOGIN_eq_125 – identified in the original study as the most similar to our form III model. However, this predicted structure does not belong to the lowest-energy structures predicted on the crystal-energy landscape. The root-mean-square deviation obtained from the overlay of 90 molecules of the two crystal structures in the *Crystal Similarity Tool* (Chisholm & Motherwell, 2005 ▸) is 0.329 Å (Fig. S11). Characterized by the same SC_0_ dimeric building block, UNOGIN_eq125 packs in the same space group *P*2_1_/*c* as form III, but the unit-cell parameters are slightly distorted (Table S4), and the calculated powder pattern differs from that of phase III at resolutions higher than 5 Å (above 18° in 2θ), as shown in Fig. S12. Apart from these differences, we can say that UNOGIN_eq125 is a good approximation of form III, but unfortunately it seems that the energy of the system is quite sensitive to small distortions, and this has hampered CSP to find the correct minimum.

## 3. Conclusions

We have successfully found the missing tile to the OLZP puzzle, determining the anhydrous form III by means of 3D ED. This crystallizes in the monoclinic space group *P*2_1_/*c* and the results were confirmed by a successful Rietveld refinement of the OLZP starting phase mixture. Form III of OLZP is characterized by the same dispersion dimer observed in phases I and II. Although very similar to form II, it shows a slightly different packing with a characteristic shift every second 2D layer. Remarkably, the structure of form III differs from all the predicted crystal structures by CSP except the one that results in a close, but not identical structure, although it does not belong to the lowest-energy group. Our results confirm once again that the use of 3D ED for characterizing elusive pharmaceutical phases is a viable solution able to solve crystallographic problems that have been unsolved for many years.

## 4. Related literature

The following references are cited in the supporting information: Ballabriga *et al.* (2020 ▸); Burla *et al.* (2015 ▸); Dolomanov *et al.* (2009 ▸); Gemmi & Lanza (2019 ▸); Macrae *et al.* (2020 ▸); Palatinus & Chapuis (2007 ▸); Sheldrick (2015 ▸); Spackman *et al.* (2021 ▸); Yang *et al.* (2022 ▸).

## Supplementary Material

Crystal structure: contains datablock(s) global, I. DOI: 10.1107/S2052252524007383/ur5003sup1.cif

Structure factors: contains datablock(s) I. DOI: 10.1107/S2052252524007383/ur5003sup2.hkl

Supporting figures and tables. DOI: 10.1107/S2052252524007383/ur5003sup3.pdf

CCDC reference: 2309728

## Figures and Tables

**Table 1:** Unit-cell parameters of forms III, II and I from the data collected with 3D ED at room temperature Crystallographic and refinement information for forms III (dynamically refined) and II (kinematically refined) are also reported.

	III	II	I
No. of indexed crystals	18	10	4
Crystal system	Monoclinic	Monoclinic	Monoclinic
Space group	*P*2_1_/*c*	*P*2_1_/*c*	–
*a* (Å)	10.708 (3)	9.9664 (41)	10.33 (5)
*b* (Å)	16.476 (4)	16.693 (1)	15.262 (3)
*c* (Å)	10.065 (4)	9.939 (1)	10.52 (1)
α (°)	90	90	90
β (°)	110.43 (2)	98.65 (1)	100.5 (6)
γ (°)	90	90	90
Volume (Å^3^)	1664	1635	1630
*Z*/*Z*′	4/1	4/1	–
Density (calc.) (Mg m^−3^)	1.25	1.27	–
*F*(000)	269	269	–
Crystal size	Nanocrystal	Nanocrystal	Nanocrystal
Theta range (°)	0.1–1.19	0.19–2.11	–
Index ranges	−12 ≤ *h* ≤ 12	−10 ≤ *h* ≤ 10	–
	−19 ≤ *k* ≤ 19	−18 ≤ *k* ≤ 17	–
	−11 ≤ *l* ≤ 11	−10 ≤ *l* ≤ 10	–
Reflections collected	16427	3054	–
Independent reflections	3274	1523	–
Completeness (%)	88	66.9	–
*I*/σ(*I*)	4.5	4.5	–
*R*_int_	19.95	18.28	–
Refinement type	Dynamical_Full-matrix non-linear least-squares	Kinematical_Full-matrix least-squares on *F*^2^	–
Data/restraints/parameters	3084/6/269	1523/0/202	–
GoF	3.2	1.4	–
*R*_1_ (%)	12.23	20.61	–
*wR*_2_ (%)	12.26	46.61	–
*R*_1_(all) (%)	20.34	26.31	–
*wR*_2_(all) (%)	13.24	51.91	–

## Data Availability

The authors confirm that the data supporting the findings of this study are available within the article and its supporting information.
