# Supplementary material for: The crystal structure of olanzapine form III
Source: IUCrJ. 2024 Jul 29;11(Pt 5):843–8. doi: 10.1107/S2052252524007383 (PMC11364035; doi:10.1107/S2052252524007383)
Supplement: Supplementary file 3 [file m-11-00843-sup3.pdf]

IUCrJ

Volume 11 (2024)

Supporting information for article:

The crystal structure of Olanzapine form III

Goulielmina Anyfanti, Elena Hasanu, Iryna Andrusenko, Danilo Marchetti and Mauro Gemmi

S1. Experimental

IUCr body text OLZP was purchased from TCI, dichloromethane was purchased from Sigma. A determined quantity of OLZP as received was completely dissolved in dichloromethane. A yellow liquid solution was immediately obtained, filtered with a 0.45 µm PTFE filter attached to a 5 ml syringe in a petri dish and let to evaporate under the hood. To assure the complete removal of the solvent, the powder obtained was transferred in a BUCHI oven at 40 °C under reduced pressure.

PXRD patterns of the sample packed in 0.5 mm borosilicate glass capillary were recorded in transmission mode at room temperature on a STOE STADI-P diffractometer (STOE, Darmstadt, Germany) equipped with a Ge (111) monochromator and a Mythen2 1K detector from DECTRIS, using Cu-Kα1 radiation (λ=1.5406 Å). The data acquisition and first analysis were processed with WinXPOW software (STOE). DSC measurements were performed with a Mettler Toledo DSC1 analyser in a N2 flow and a heating rate of 10°C/min.

Le Bail intensities extraction and Rietveld refinements of the powder mixture were performed in Jana2020. (Petříček *et al.*, 2023)

Microcrystals from the powder mixture were deposited on 300-meshed copper grids coated with carbon. Electron diffraction experiments were performed on a Zeiss Libra 120 TEM operating at 120 kV and equipped with an LaB6 thermionic source and an in-column omega filter. Data were obtained in continuous rotation mode as reported in the paper by Gemmi & Lanza. (Gemmi & Lanza, 2019) During the data collection the crystal position was tracked in high magnification scanning transmission electron microscopy (STEM) imaging as proposed by Yang et al. (Yang *et al.*, 2022). Diffraction patterns were collected in nanodiffraction mode with a parallel beam of 150 nm obtained using a 5 µm condenser aperture. 3D ED diffraction data were recorded using an ASI Timepix detector. (Ballabriga *et al.*, 2020) Peak indexing and intensity integration were performed using Pets2. (Palatinus *et al.*, 2019) Space group determination and structure solution for phase III were obtained with Superflip (Palatinus & Chapuis, 2007) in Jana2020. (Petříček *et al.*, 2023) Structure of form II was solved by directs methods in Sir2019. (Burla *et al.*, 2015) Dynamical refinements for form III were performed against two data set of two different crystals at a resolution of 1 Å. The lattice parameters of crystal 2, therefore, were fixed to be the same as those of crystal 1. The data sets collected from crystals 1 and 2 were kept in two data blocks (Block 1 and Block 2 respectively) and a combined refinement against the two blocks was performed in Jana2020. (Petříček *et al.*, 2023) The processed data set for continuous rotation was refined with the thick model wedge (Palatinus *et al.*, 2015). The orientation of each pattern was optimized, and the ADPs were refined as anisotropic (ADPs of hydrogen were kept anisotropic with the riding model on). In the thick model wedge the crystal is assumed to be wedge shaped. Structure of form II was kinematically refined with SHELXL (Sheldrick, 2015) in Olex2 GUI. (Dolomanov *et al.*, 2009)

Molecular graphics were created with Mercury 4.0. (Macrae *et al.*, 2020) The repository of predicted crystal structure for OLZP was provided by the authors and a screen was performed using the Crystal Packing Similarities (CPS) module provided in Mercury software.

The Hirshfeld surfaces were generated using the CrystalExplorer software (version 21.5) at very high resolution. (Spackman *et al.*, 2021)

S2. Le Bail intensity extraction of OLZP

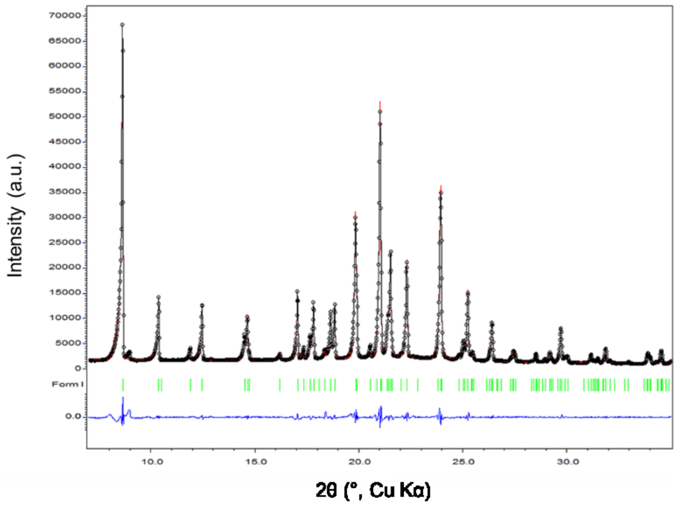

Figure S1 Le Bail fit of OLZP purchased from TCI®. The refined cell dimensions taken from form I (Wawrzycka-Gorczyca *et al.*, 2004) are a = 10.39821(14) (Å), b = 14.86624(14) (Å), c = 10.57299(12) (Å), β = 100.6391(19)˚, R_{obs} = 3.12%, wR_{obs} = 4.72%.

S3. PXRD and DSC of the multiphase mixture

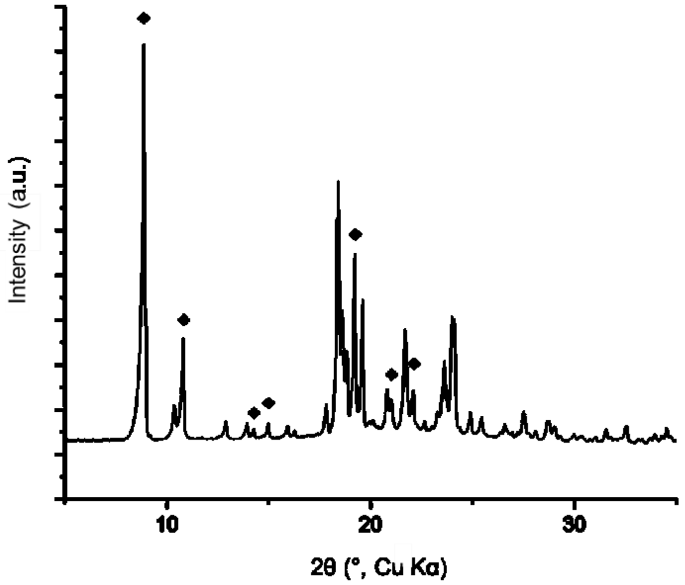

Figure S2 PXRD pattern of the multiphase mixture obtained by recrystallisation of purchased OLZP in dichloromethane. The peaks that testify the presence of multiple phases are signed with the black rhomboidal symbol and correspond to what observed by Bhardwaj *et al*. (Bhardwaj *et al*., 2013)

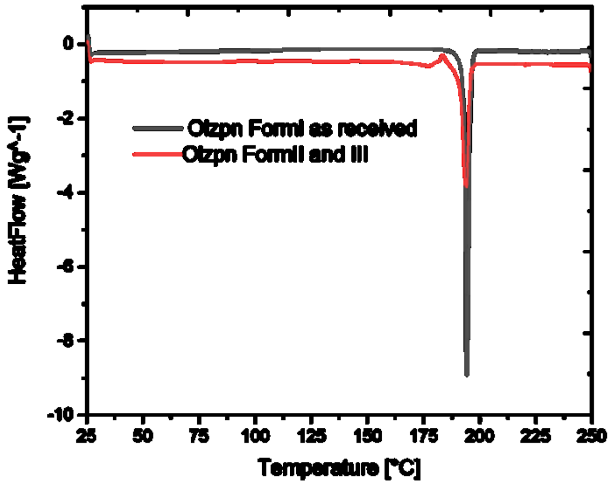

Figure S3 DSC thermograms of powder OLZP form I as received (black line) and the mixture of form II and III (red line) obtained at a flow rate of 10°C/min between 25°C and 250°C upon heating.

S4. Crystallographic images and information of forms III, II and I of OLZP

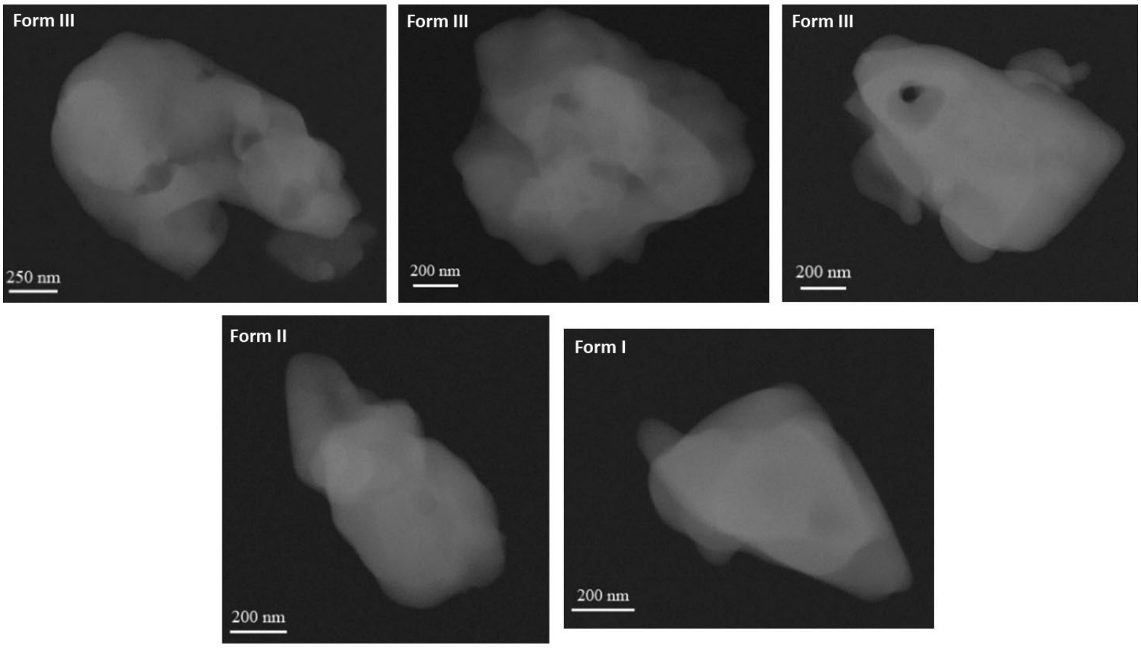

Figure S4 STEM images of OLZP nanocrystals characteristic of form I, form II and III. The images were acquired using an HAADF detector.

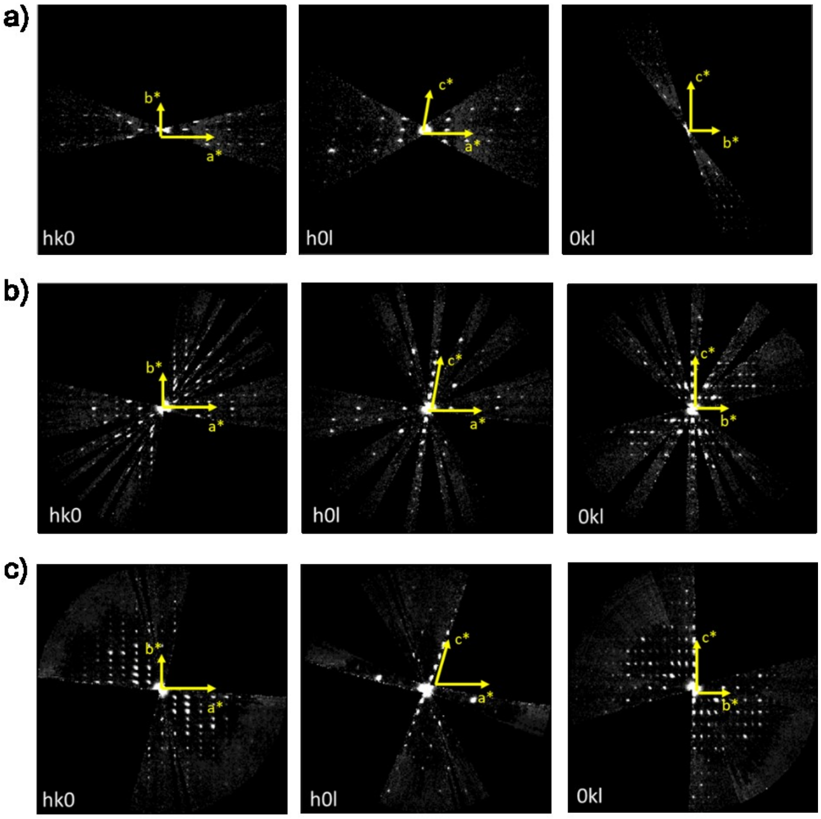

Figure S5 Images of the reciprocal sections projections hk0, h0l and 0kl respectively of phase I (a), phase II (b) and III (c).

Table S1 Crystallographic information and dynamical refinement results of structure form II.

Crystallographic Information	
Crystal system	monoclinic
Space group	$P2_1/c$
a (Å)	9.9664/41)
b (Å)	16.693(1))
c (Å)	9.9938(1)
α (°)	90
β (°)	98.65(1)
γ (°)	90
Volume ($Å^3$)	1635
Z / Z'	4 / 1
Density (calc.) (Mg m^{-3})	1.25
F(000)	268
Crystal size	nanocrystal
Theta range (°)	0.19-2.11
Index ranges	$-10 \leq h \leq 10$<br>$-18 \leq k \leq 17$<br>$-10 \leq l \leq 10$
Reflections collected	6860
Independent reflections	2705
Completeness (%)	85
I/sig(I)	3.3
R_{int}	15.05
Type of refinement	Full-matrix non-linear least-squares
Data/restraints/parameters	2705/20/195
GoodF	3.76
R_1 (%)	14.2
wR_2 (%)	14.2
R_1(all) (%)	23.5
wR_2(all) (%)	15.2

Table S2 Crystallographic information of the kinematical refinements results performed on the structure of form III obtained from the data collected with 3D ED from crystal 1 (first column), crystal 2 (second column) and from the merged data of crystal 1 and crystal 2.

	Crystal 1	*Crystal 2*	*Merged Data*
Crystal system	monoclinic	monoclinic	monoclinic
Space group	$P2_1/c$	$P2_1/c$	$P2_1/c$
a (Å)	10.828	10.594	10.611
b (Å)	17.01	16.576	16.422
c (Å)	9.759	10.075	10.161
α (°)	90	90	90
β (°)	109.47	109.93	110.29
γ (°)	90	90	90
Volume (Å^3)	1694.6	1663.3	1660.6
Z / Z'	4 / 1	4 / 1	4 / 1
Density (calc.) (Mg m^{-3})	0.655	0.667	1.25
F(000)	271	271	261
Crystal size	nanocrystal	nanocrystal	nanocrystal
Theta range (°)	0.22-1.918	0.192-1.918	0.192-1.958
	$-10 \leq h \leq 10$	$-10 \leq h \leq 10$	$-10 \leq h \leq 10$
Index ranges	$-16 \leq k \leq 16$	$-16 \leq k \leq 10$	$-16 \leq k \leq 16$
	$-9 \leq l \leq 9$	$-7 \leq l \leq 7$	$-10 \leq l \leq 10$
Reflections collected	2395	2667	4726
Independent reflections	1239	1122	1695
Completeness (%)	70	65	91.8
I/sig(I)	4	4.3	4.5
R_{int}	0.14	0.23	0.2479
Type of refinement	Full-matrix least-squares on F2	Full-matrix least-squares on F2	Full-matrix least-squares on F2
Data/restraints/parameters	1239/245/202	1122/0/91	1695/184/202
GoodF	1.683	1.573	1.524
R_1 (%)	0.26	0.24	0.2183
wR_2 (%)	0.55	0.51	0.5002
R_1(all) (%)	0.31	0.29	0.2754
wR_2(all) (%)	0.6	0.55	0.5407

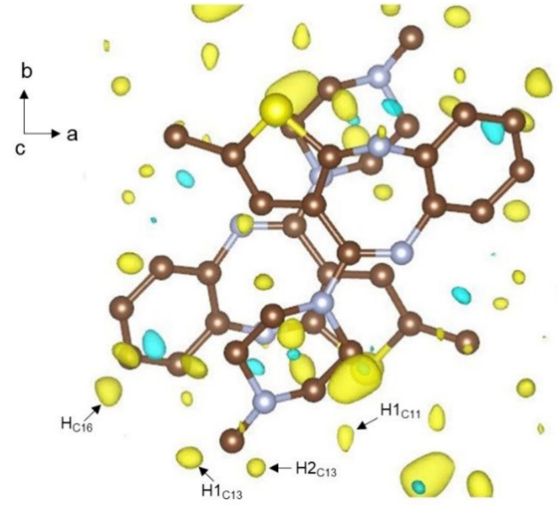

Figure S6 Difference Fourier map of OLZP form III before adding hydrogen atoms calculated at a 3σ isosurface level. Just a few hydrogen atoms can be seen. Positive and negative isosurfaces are plotted in yellow and blue, respectively.

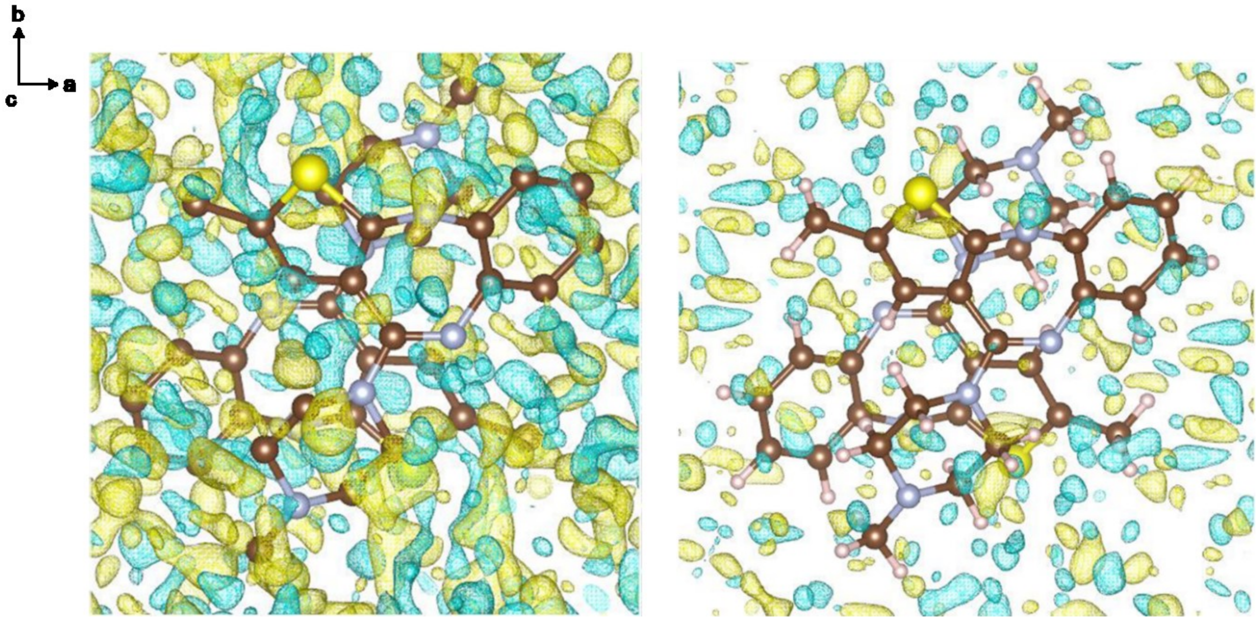

Figure S7 The two difference Fourier map of OLZP form III calculated at the same isosurface value of 0.27 eÅ^{-1} before (left) and after (right) adding the expected hydrogen atoms viewed along the c-axis direction. Positive and negative isosurfaces are plotted in yellow and blue, respectively.

S5. Le Bail intensity extraction and Rietveld refinements results of the multiphase mixture

The unit cell parameters of forms I were not refined due to its little content. The refined cell dimensions after Le Bail extraction intensities are: for form II a = 9.933914(30) (Å), b = 16.54212(40) (Å), c = 10.01167(28) (Å), β = 98.06863(35)˚; for the new form III they are a = 10.61363(19) (Å), b = 16.47721(28) (Å), c = 10.02741(26) (Å), β = 110.2544(21)˚. The agreement factors of the fit are R_{obs} = 1.90%, wR_{obs} = 2.84%. In the case of Rietveld refinement, the patterns were modelled with a mixture of forms III, II and I in infinitesimal quantity. The unit cell parameters of the latter were not refined. The refined cell dimensions and refinement parameters for form III and II are respectively a = 10.61457(34) Å, b= 16.47859(43) Å, c= 10.02385(30) Å, β = 110.2624(28)˚ and a = 9.93127(53) Å, b= 16.5275(66) Å, c= 10.01208(64) Å, β = 98.0086(57)˚. The relative agreement factors are R_{obs} = 3.05% and wR_{obs} = 4.25% for form III, R_{obs} = 3.40% and wR_{obs} = 4.51% for form II, R_{obs} = 3.28% and wR_{obs} = 3.65% for form I.

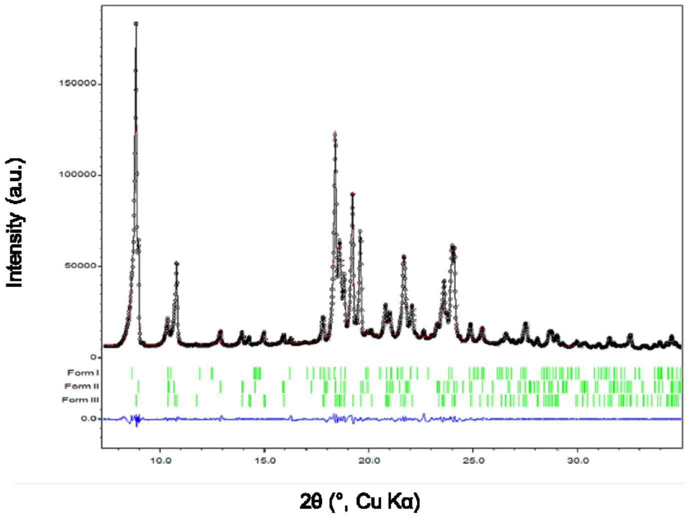

Figure S8 Le bail intensities extraction of the mixed forms I, II and III of OLZP obtained after recrystallisation.

S6. 2D-finger prints of Hirshfeld surfaces of forms II and III

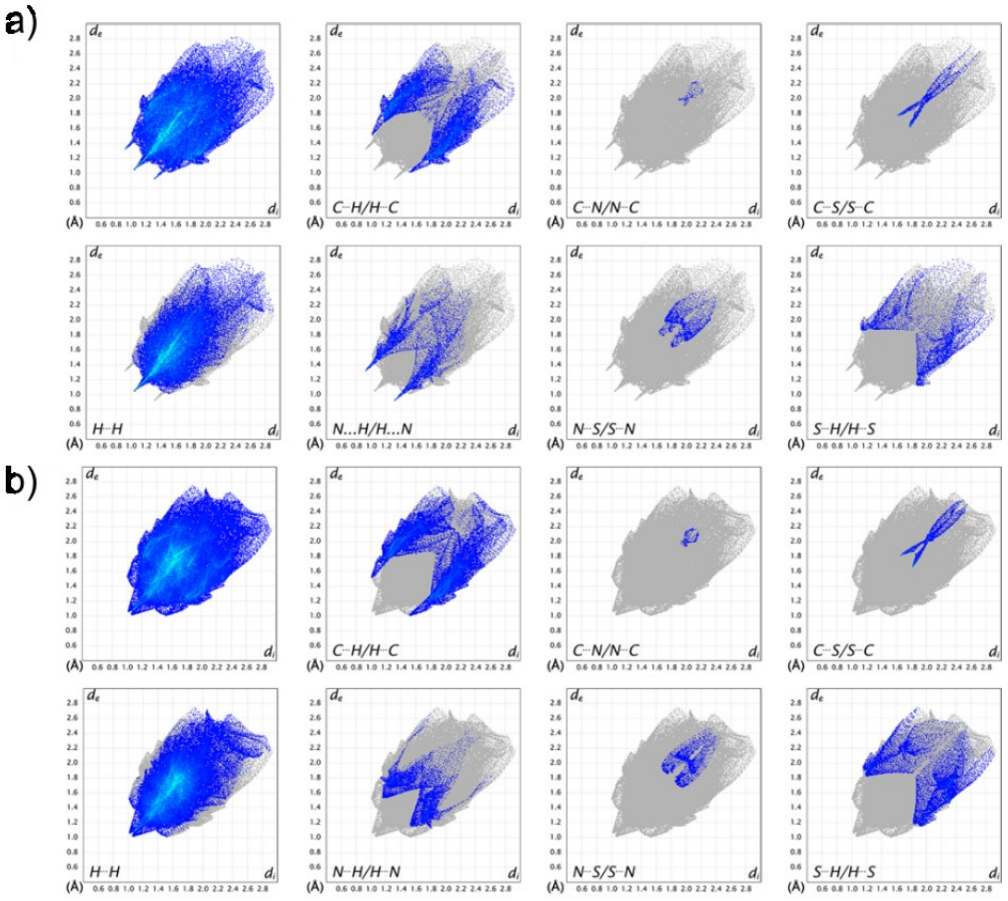

Figure S9 Comparison between fingerprint plots for the single molecule of OLZP in form II (A) and form III (B).

Table S3 Percentage contributions to the Hirshfeld surface area of the different close intermolecular contacts of OLZP molecule in forms II and III.

Contact Type	% in Form II	% in Form III
C…H/H…C	21.9	20.2
N…H/H…N	7.2	5
S…H/H…S	6	7.1
N…S/S…N	2.1	1.5
C…S/S…C	1.9	1.9
C…N/N…C	0.2	0.3
H…H	60.7	63.8

S7. Comparison of A162 with form II

Figure S10 (top) shows the packing of two layers (green and pink) of the structure A162 viewed along the relative b-axis direction. Hiding the pink layer and viewing the structure along [-1 2 0] direction what we see is the equivalent of the green layer encountered in forms II and III (fig. S10 bottom left). On the other hand, omitting the green layer and viewing the structure along the [1 2 0] direction (fig. S10 bottom right) the pink layer appears shifted of one half along the bc-plane, but the presence of the glide plane parallel to the (001) and normal to the (010) causes the exchange of the SC_0 enantiomers every second layer.

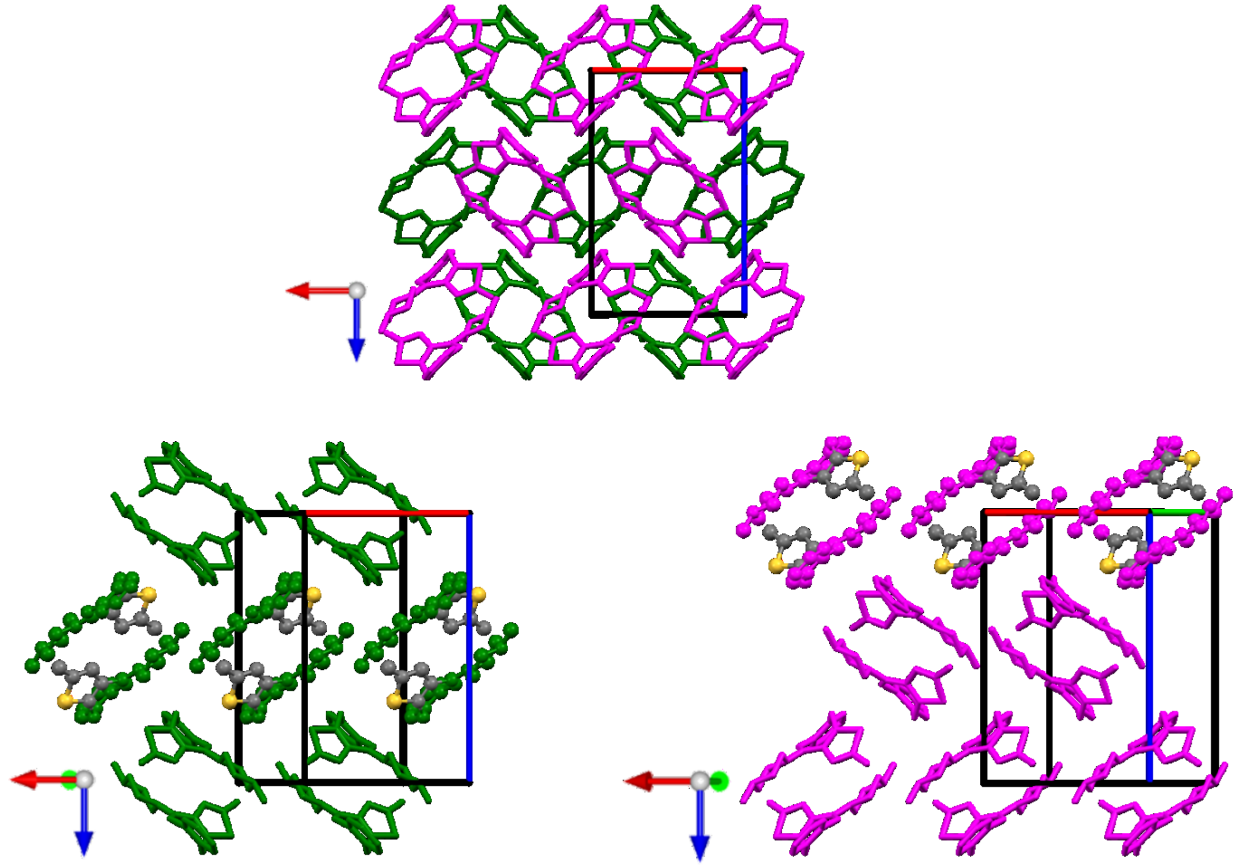

Figure S10 (Top) Packing of two layers in structure A162 viewed along the b-axis; (bottom left) green equivalent layer viewed along the [-1 2 0] direction and (bottom right) the pink layer viewed along the [1 2 0] direction. The unit cell axis are coloured in red (a-axis), green (b-axis) and blue (c-axis).

S8. Comparison of UNOGIN_eq125 with phase III

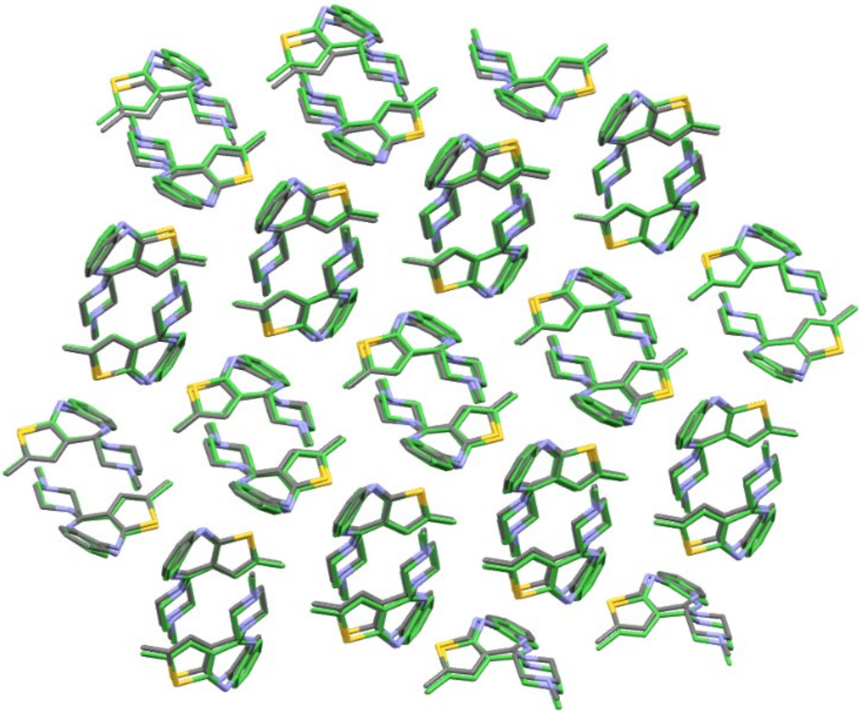

Figure S11 Overlay of the experimental form III (coloured by element with C grey, N light blue, and S yellow) with UNOGIN_eq125 (green).

Table S4 Unit cell parameters of OLZP form III and the predicted structure UNOGIN_eq125.

Polymorph	III	UNOGIN_eq125
a (Å)	10.71(5)	11.7
b (Å)	16.48(7)	16.9
c (Å)	10.07(5)	10.2
α (°)	90	90
β (°)	110.4(2)	58.4
γ (°)	90	90
V ($Å^3$)	1664	1717

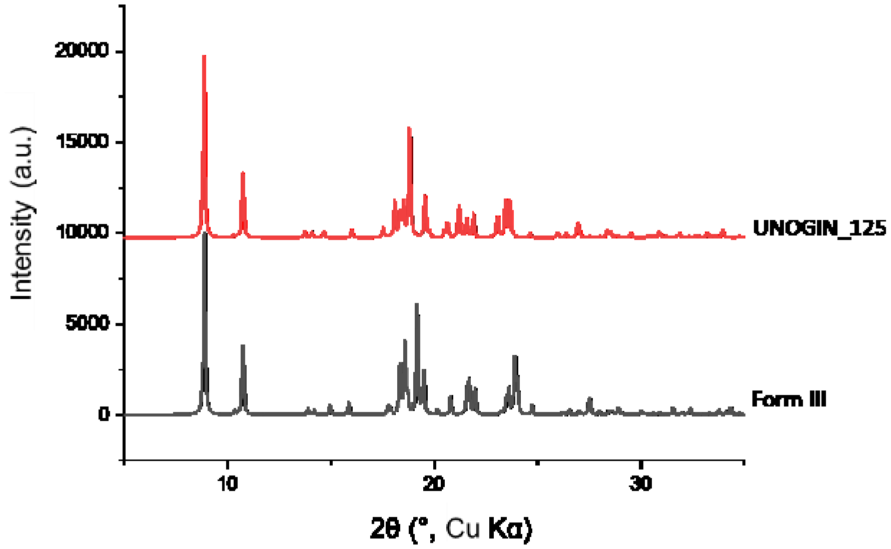

Figure S12 Comparison of the calculated PXRD patterns of phase III (black) with the predicted structure UNOGIN_eq125. The two graphs differ especially between the 2θ range of 15° and 25°.
